# Serological Evidence of Exposure to EBHSV and RHDV2 in Brown Hare (*Lepus europaeus* Pall. 1778) in Poland: Insights into Lagovirus-Host Interaction and Population Dynamics

**DOI:** 10.3390/biology15211934

**Published:** 2026-10-25

**Authors:** Marian Flis, Krzysztof Łazowski, Piotr Czyżowski, Beata Hukowska-Szematowicz, Natalia Brodalka, Andrzej Fitzner

**Affiliations:** 1 Department of Animal Ethology and Wildlife Management, Faculty of Animal Sciences and Bioeconomy, University of Life Sciences in Lublin, Akademicka 13, 20-950 Lublin, Poland; marian.flis@up.edu.pl; 2 Polish Hunting Association, District Board in Tarnów, 300 Sieradza, 33-240 Żabno, Poland; krislex@poczta.fm; 3 Laboratory of Infectious Biology, Molecular Biology and Immunology, Institute of Biology, University of Szczecin, Wąska 13, 71-415 Szczecin, Poland; beata.hukowska-szematowicz@usz.edu.pl; 4 Molecular Biology and Biotechnology Center, University of Szczecin, Wąska 13, 71-415 Szczecin, Poland; 5 National Veterinary Research Institute—State Research Institute, Department of Virology and Viral Animal Diseases, FMD Research Team, 98-220 Zduńska Wola, Poland; natalia.brodalka@piwet.pulawy.pl; 6 National Reference Laboratory for Rabbit Hemorrhagic Disease (RHD), National Veterinary Research Institute—State Research Institute, Wodna 7, 98-220 Zduńska Wola, Poland

**Keywords:** *Lepus europaeus*, population indicator, animal viruses, European brown hare syndrome (EBHSV), rabbit haemorrhagic disease virus 2 (RHDV2), host-pathogen interaction, seroprevalence

## Abstract

The long-term decline in European hare populations across Europe is linked to both environmental and anthropogenic factors. Among the factors influencing the population dynamics of lagomorphs, lagoviruses play a significant role. This study evaluated population indices and the presence of antibodies against EBHSV and RHDV2 in hares in Poland. A total of 109 individuals were examined from five regions where population density allowed for game management. Population indices were assessed, and the presence of antibodies was determined using ELISA tests with monoclonal antibodies. Body mass of hare was similar across all regions but differed significantly between juveniles and adults. Population growth rates were positive, while reproductive rates remained relatively low. Antibodies specific to EBHSV in hare were detected in all five regions—with an overall seroprevalence of 78.9%—indicating widespread prior exposure to this virus among the hares studied. RHDV2-reactive antibodies were found in 10.1% of the individuals. Given the risk of cross-reactivity and individual immune responses, results regarding RHDV2 should be regarded as serological evidence of possible exposure requiring further investigation. The findings provide serological evidence of lagovirus exposure in the examined hare populations even in areas with population densities sufficient for game management. These results underscore the importance of integrated hare population monitoring that combines traditional population metrics with serological surveillance, particularly regarding lagoviruses such as EBHSV and RHDV2.

## Simple Summary

European hares face various environmental pressures that shape their population dynamics and health. This study analyzed 109 individuals from five regions in Poland during the 2022/2023 hunting season to evaluate population parameters like body mass and age structure, while also screening for lagoviruses. The results showed that while population growth rates were positive, a low reproduction index signals potential challenges for recovery. Furthermore, serological tests revealed a high prevalence of European brown hare syndrome virus (EBHSV) antibodies at 78.9% (95% CI: 70.1–86.1%), indicating widespread prior exposure to this pathogen in all the regions analyzed. In addition, RHDV2-reactive antibodies were detected in 10.1% (95% CI: 5.2–17.3%) of the examined hares in some of the studied habitats, although possibility of cross-reactivity with EBHSV cannot be excluded. Ultimately, combining demographic monitoring with pathogen surveillance is crucial for understanding how lagoviruses influence wild hare populations and conservation efforts.

## 1. Introduction

The European hare, or brown hare (*Lepus europaeus*), is one of the most characteristic representatives of the order of lagomorphs and was, until recently, widespread throughout Europe, including Poland. This species plays a significant role in the functioning of agricultural and open ecosystems, forming an important part of food webs and serving as an indicator of environmental condition. However, since the late 1960s, a downward trend in the population of this species has been observed in most European countries [1,2,3,4,5]. This trend has also affected Poland, where local population densities are currently lower than the annual harvest levels recorded in the 1960s and 1970s [6,7,8,9]. Nevertheless, population densities remain sufficiently high in some areas to permit sustainable annual harvesting through regulated hunting [10,11,12]. It is worth noting that hunting in areas with high population densities does not directly affect population growth dynamics, as it is compensatory, removing only the surplus and thus reducing population density in line with the decline in resource availability before winter [13].

Although it is not possible to point to the specific causes of such a dramatic decline in the hare population, the main factors cited are the intensification of agriculture, which, combined with the widespread use of plant protection products and climate change, has simplified agro-community structures and, consequently, led to the loss of habitats for this species [1,14]. This was confirmed by a study conducted in Austria, which showed a significant increase in hare survival rates depending on the proportion of fallow land compared with cultivated land [15]. In turn, climatic factors contribute to population decline, particularly among juvenile individuals, for whom the mortality rate in the first month of life was approximately 18% [16]. Predator pressure, mainly posed by foxes, also plays a significant role; this is linked to the oral vaccination programmes for wild foxes that have been carried out in many European countries since the 1970s as part of rabies control efforts [17,18,19,20,21,22,23].

Diseases with different aetiologies are another factor that substantially reduces hare populations. Until recently, coccidiosis and other parasitic diseases were considered the most significant [24,25,26,27]. However, in the 1980s, a new clinical entity was diagnosed and described as the European brown hare syndrome (EBHS). The disease was first diagnosed in 1980 in Scandinavia, although evidence suggests it may have existed earlier [28]. Originally, it was thought to be a toxic-metabolic syndrome. However, because the disease spread rapidly throughout Europe and contributed to the decimation of the hare population, research has shown that it is of viral origin [29]. In the mid-1990s, following the rapid spread of the highly lethal and contagious EBHS virus (GII.1) throughout many European countries, a dramatic decline in wild hare populations was recorded [30,31,32,33,34,35,36,37]. Furthermore, in the 1980s, a similar severe viral disease in rabbits (RHD) was diagnosed, caused by a different but closely related virus from the *Caliciviridae* family, genus *Lagovirus,* known as RHDV (GI.1). The two lagoviruses share numerous clinical, pathological and epizootic similarities [38,39]. The structural and evolutionary similarities between the viruses described are confirmed by cross-reactive immune responses [40]. In Poland, the first EBHSV isolate was detected in hares in 1992 [41,42]. During the same period, the presence of the virus was also confirmed in other studies conducted in western Poland. These studies also confirmed, for the first time, the presence of anti-RHDV antibodies in 3% of native hares [43]. In the early 2000s, outbreaks of EBHS were reported in Poland among hares kept in captivity, and genetic analyses revealed a close relationship between Polish and European strains of EBHSV [44]. In 2010, a new type of rabbit haemorrhagic disease virus, RHDV2 (GI.2), which causes the death of vaccinated rabbits and juvenile rabbits under one month of age, was detected in France [45,46]. RHDV2 strains (also referred to as RHDVb) were also isolated in 2011 in Spain [47]. The study showed that disease caused by this virus differs in duration, mortality rates and the higher prevalence of the subacute form, as well as partial cross-protection between RHDV and RHDV2. RHDV2, which affects both farmed and wild rabbits, is currently detected almost worldwide and can cause a disease resembling EBHS in various hare species. It has also been reported in species other than lagomorphs [48,49,50,51,52]. Another study covering two regions of Poland, conducted between 2020 and 2021, detected no RHDV2 in the 113 hares tested [53].

The aim of this study was to assess selected population indicators and the presence of antibodies against EBHSV and RHDV2 in brown hares (*Lepus europaeus*) from selected regions of Poland where population densities allow for sustainable game management. Exposure to both lagoviruses was assessed based on the presence of antibodies against EBHSV and RHDV2, detected using highly specific ELISA tests. By integrating population indicators with serological findings, the study aimed to provide information on lagovirus exposure in free-ranging brown hare populations.

## 2. Materials and Methods

### 2.1. Animals

**Figure 1:**
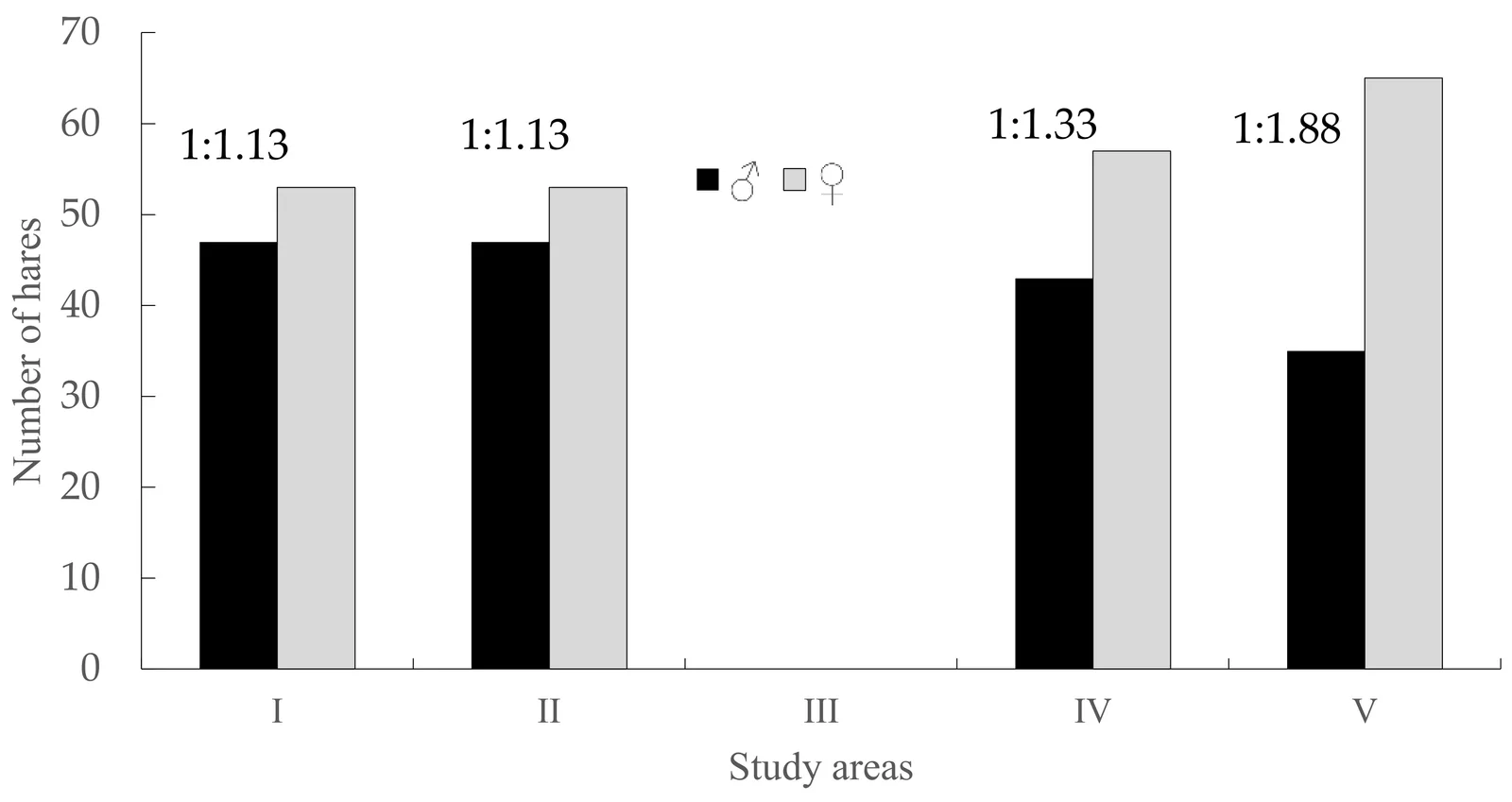
Hare sex structure in research areas (expressed as a ratio of males to females).

**Scheme 1:**
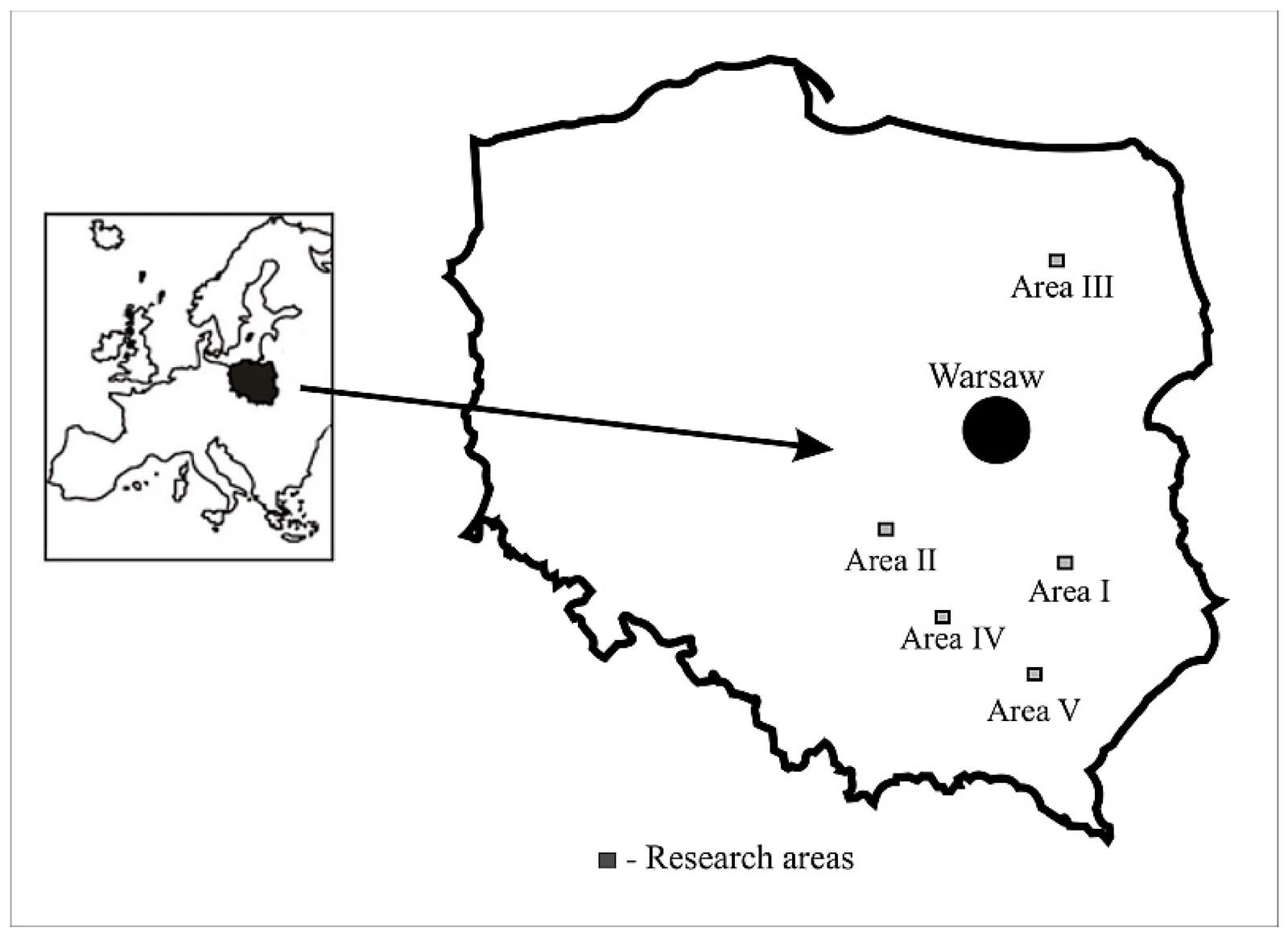
Spatial distribution of the research areas.

The study was conducted on a sample of 109 hares harvested in five regions of eastern Poland (Scheme 1 and Figure 1). As the biological material for the study was collected from dead hares obtained in accordance with Polish hunting legislation, no approval from the local ethics committee was required to carry out the study. The hares were harvested during the 2022/23 hunting season. Immediately after harvesting, body mass was measured using a portable KERN HCB20K10 scale (KERN & SOHN GmbH, Balingen, Germany). The hares’ age was determined by initially categorising them into juveniles or adults based on body mass [54], and then refined using the method proposed by Stroh. This method classifies individuals as juveniles or adults based on the presence or absence of cartilaginous swelling on the forelimb [55]. This enabled distinguishing between juveniles (up to 1 year old) and adults (over 1 year old). In turn, sex was determined on the basis of the presence of secondary sexual characteristics [7]. Blood samples were collected in the field directly from harvested hares for analysis.

### 2.2. Population Indicators

To describe population growth dynamics, the coefficient of reproduction, reproductive index/success rate, coefficient of population increase and population increase were used [3].

Coefficient of reproduction:

(1) $$W_{r}=\frac{N_{\mathrm{juv}}}{N_{\mathrm{ad}}}$$

Reproductive index/success rate:

(2) $$W_{\mathrm{sr}}=\frac{N_{\mathrm{juv}}}{N_{\mathrm{adf}}}$$

Coefficient of population increase:

(3) $$W_{\mathrm{pp}}=0.7(\frac{N_{\mathrm{juv}}}{N_{\mathrm{ad}}}+1)$$

Population increase:

(4) $$W_{p}=\frac{{\%}_{\mathrm{juv}}-30}{{100-\%}_{\mathrm{juv}}}\times 100$$

where:

N_juv_—number of juveniles

N_ad_—number of adults

N_adf_—number of adult females

%_juv_—percentage of juveniles

Furthermore, the sex and age structure of hares in each research area was calculated.

### 2.3. Serological Methods

Antibodies against EBHSV and RHDV2 in hare blood serum samples were detected using two ELISA kits for EBHSV and RHDV2 serology, commercially available from the Istituto Zooprofilattico Sperimentale della Lombardia e dell’Emilia (IZSLER) in Brescia, Italy (IZSLER product codes: 72574 and 78751). The tests were carried out in accordance with the manufacturer’s instructions. Each ELISA kit contains appropriate negative and positive control sera, viral antigen and specific monoclonal antibodies conjugated to horseradish peroxidase, enabling highly sensitive detection of antibodies against EBHSV and RHDV2. The serum titre corresponds to the dilution of the test serum that inhibits the absorbance (492 nm) of the negative control serum by 50%.

### 2.4. Statistical Analysis

In the database, variables relating to serological status (the presence of antibodies against EBHSV and RHDV2-reactive antibodies) were encoded in a binary system as dichotomous variables (‘0’—negative result—no antibodies or a titre below 10; ‘1’—positive result/presence of antibodies with a titre of 10 or higher), while antibody titres were entered as continuous values.

The normality of the distribution of the continuous variables analysed was verified using the Shapiro–Wilk test. The homogeneity of variances in the groups being compared was assessed using Levene’s test. To assess the significance of differences between two independent groups for normally distributed variables, the t-test for independent samples was employed. When analysing antibody titres, the non-parametric Mann–Whitney U test was used because the assumptions of normality were not met. An analysis of the prevalence of antibodies (seroprevalence) by gender and age was carried out using Fisher’s exact two-sided test and the chi-squared test of independence. Differences in seroprevalence and antibody titres between the individual geographical regions (REGs 1–5) were assessed using the Kruskal–Wallis test.

A multifactorial assessment of the determinants of seroprevalence was carried out using generalised mixed-effects models of analysis of covariance (Mixed ANCOVA). In these models, the region of origin (REG) was included as a random effect; sex, age and (in the model for RHDV2) EBHSV serological status were included as fixed effects, while the individual’s body mass was included as a covariate.

To account for the hierarchical data structure while managing binary outcomes in an exploratory context, we applied linear mixed-effects models as an exploratory linear probability framework, treating regional variability as a random effect.

All statistical calculations and graphical visualisations were performed using Statistica 13.1 software with a standard significance level of α = 0.05.

## 3. Results

### 3.1. Structure of the Populations Under Assessment

**Figure 2:**
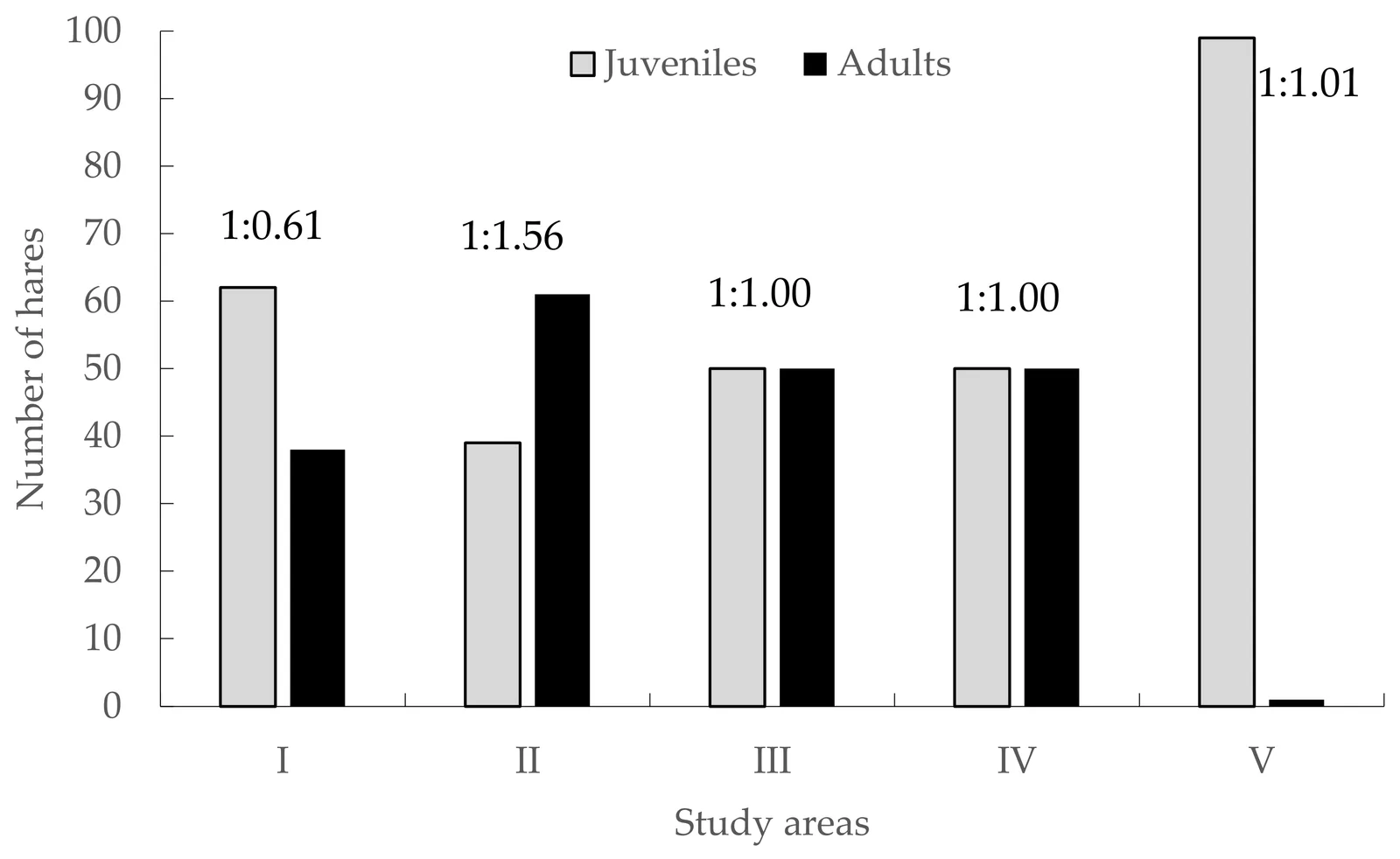
Hare age structure in research areas (expressed as a ratio of juveniles to adults).

The body mass of both juvenile and adult hares showed little variation between the areas from which the animals originated (Table 1). No statistically significant difference in body mass was detected between males (4.09 kg) and females (4.11 kg) in the study sample (t = −0.381; *p* = 0.704; Levene’s test: *p* = 0.538). In contrast, body mass differed significantly between age groups (t = −10.212; *p* < 0.001; Levene’s test: *p* = 0.252). Adult hares (A) were, on average, more than 0.5 kg heavier (4.40 kg) than juvenile hares (J) (3.88 kg). Overall, the study population showed a higher proportion of females (56%) than males (44%), and juveniles (57.8%) were more common than adult hares aged over one year (42.2%) (Figure 1 and Figure 2). However, the percentage share of the above-mentioned categories (age/sex) in individual groups of animals deviated from the average values, as was the case in Area III, where adult hares accounted for 100%, and in Area V, where juvenile hares accounted for 95.7% of the animals. In Region V, the extreme population growth value reflects a hunting bag composition bias (95.7% of harvested hares were juveniles) rather than a true biological rate of population recruitment.

An analysis of population indicators revealed substantial variation in the parameters assessed across the individual research areas (Table 1). Nevertheless, the coefficient of population growth was positive in all areas, and its values indicate that these populations are growing at a moderate rate. The values of the coefficient of reproduction are rather concerning, as it fluctuated around 1.0 (except for Area V), indicating a decline in the condition of individual populations in terms of their potential for further growth. However, the small sample sizes do not allow any clear conclusions to be drawn in this regard.

### 3.2. EBHSV Seroprevalence

The overall seroprevalence of antibodies against the EBHSV (GII.1) in 109 wild hares from five different geographical regions of Poland, collected between November and the end of December 2022, was estimated at 78.9% (95% CI: 70.1–86.1%) (Table 2). The antibody titres recorded by the EBHSV ELISA test in 86 hares fell within the low (10–40) or medium (40–160) ranges. No antibodies against EBHSV were detected in 23 animals (21.1%). Within individual sample batches, the proportion of seropositive hares ranged from 72.3 to 87.0%. The overall percentage of EBHSV-seropositive hares was higher in females than in males, averaging 47.7% compared with 31.2%. In individual batches, the proportion of females with EBHSV antibodies among all positive individuals in the group ranged from 55.9 to 70%, while for males, it ranged from 30.0 to 45.5%. Considering the animals’ age, the proportion of EBHSV-seropositive hares was higher among juveniles than among adults, at 44% and 34.9%, respectively.

An overall assessment of the entire study sample showed that, by sex, the prevalence of EBHS infections was 85.2% (52/61, 95% CI: 73.8–93.8%) among females and 70.8% (34/48, 95% CI: 55.9–83.0%) among males. Although a higher seroprevalence was observed in females, this difference did not reach the threshold for statistical significance, showing only a weak trend (two-tailed exact Fisher’s test, *p* = 0.097; chi-squared test = 3.35, *p* = 0.067). An analysis of antibody titres using the Mann–Whitney U test showed no statistically significant differences between males and females (U = 1335.0; Z = 0.78; *p* = 0.413).

Meanwhile, analysis by sex group showed that seroprevalence of EBHSV among juvenile individuals (J) was 81.0% (51/63), while among adults (A), it was 76.1% (35/46).

No statistically significant association between age group and EBHSV seroprevalence was detected (two-tailed Fisher’s exact test, *p* = 0.482; chi-squared test, χ^2^ = 0.66, *p* = 0.417). Similarly, the comparison of antibody titers between juvenile and adult hares using the Mann–Whitney U test revealed no statistically significant difference (U = 1391.0; Z = −0.37; *p* = 0.712). Thus, the present analysis did not detect a statistically significant association between age group and EBHSV seroprevalence or a statistically significant difference in antibody titres between the two age groups.

An analysis of infection prevalence according to the animals’ area of origin showed no significant geographical differences in the seroprevalence of the EBHSV. With regard to EBHSV, the proportion of seropositive individuals across the individual areas was similar (chi-square test = 2.87; *p* = 0.580). A comparison of antibody titres across research areas, conducted using the Kruskal–Wallis test, revealed no statistically significant differences (H = 0.94; *p* = 0.918). The level of the immune response in hares was similar and independent of the source location.

**Figure 3:**
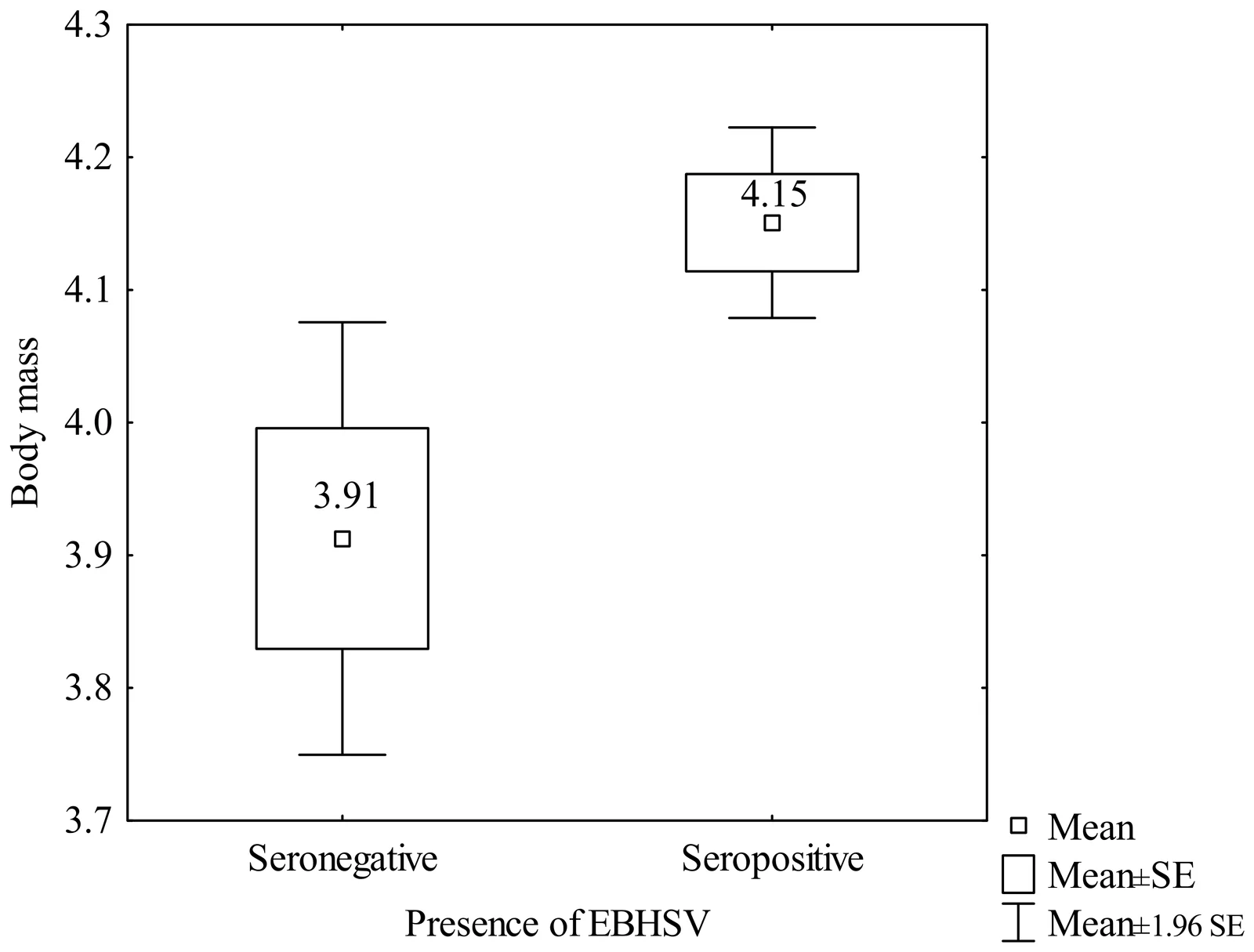
Body mass of hares according to their EBHSV serological status (seronegative, seropositive). The small squares denote mean values, the boxes represent mean ± SE, and the whiskers denote mean ± 1.96 SE.

A comparative analysis of hare body mass by EBHSV serostatus showed that EBHSV-seropositive individuals (N = 86) had a significantly higher mean body mass (4.15 kg) than EBHSV-seronegative individuals (N = 23; 3.91 kg). This difference, as determined by a t-test for independent samples, was statistically significant (t = 2.875; *p* = 0.0049). The higher average body mass of seropositive individuals (4.15 kg) compared with seronegative ones (3.91 kg) reflects the relationship between a hare’s biological maturity and the duration of its exposure to the pathogen (Figure 3).

### 3.3. RHDV2 Seroprevalence

**Figure 4:**
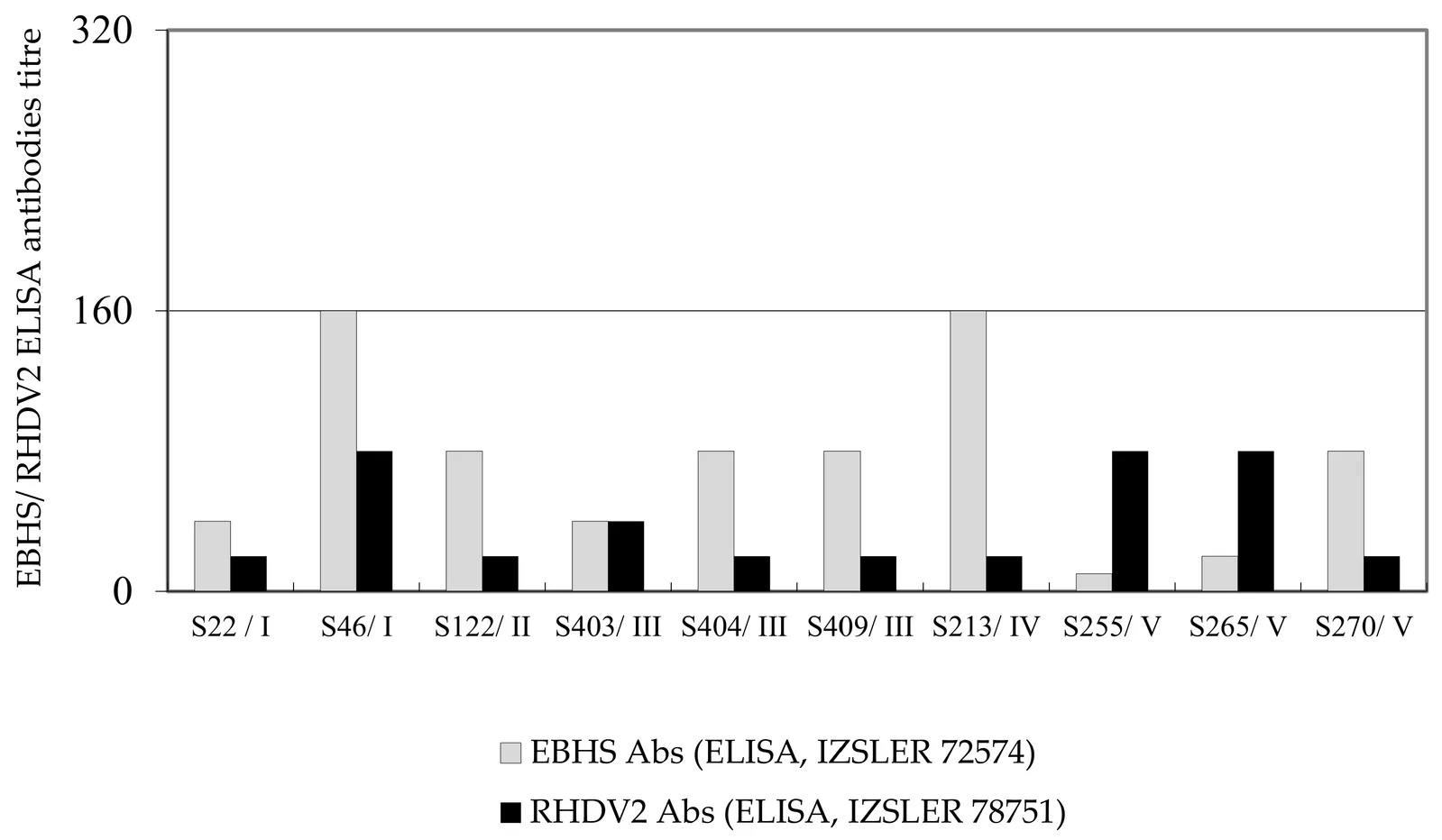
Serological test results for EBHSV antibodies and RHDV2-reactive antibodies using ELISA (titres in individual hares across the study areas; the horizontal line indicates the cut-off threshold of 160).

RHDV2-reactive antibodies were detected in 11 hare serum samples from all batches, collected in 5 research areas. The average RHDV2 seroprevalence was 10.1% (95% CI: 5.2–17.3%). The ELISA titres for RHDV2 ranged from 20 in 7 samples, through 40 in 1 sample, to 80 (the maximum value) in 3 samples (Table 3). Nine of the eleven hares that tested positive in the EBHSV ELISA (HRP conjugated with specific monoclonal antibodies) showed higher titres than those obtained in the RHDV2 serology ELISA (Figure 4). In contrast, the antibody titres in the ELISA test for RHDV2 were higher in two of the eleven hares than in the EBHS serological test. In two hares examined, the titre of RHDV2-reactive antibodies was 80, and these were the only hares in this group that, according to the interpretation by Velarde et al. [56], also applied in other studies [57], had Rt values in the RHDV2/EBHSV ELISA tests of 8 and 4, respectively, which indirectly may be interpreted as serological indication of prior exposure to the RHDV2 antigen.

**Figure 5:**
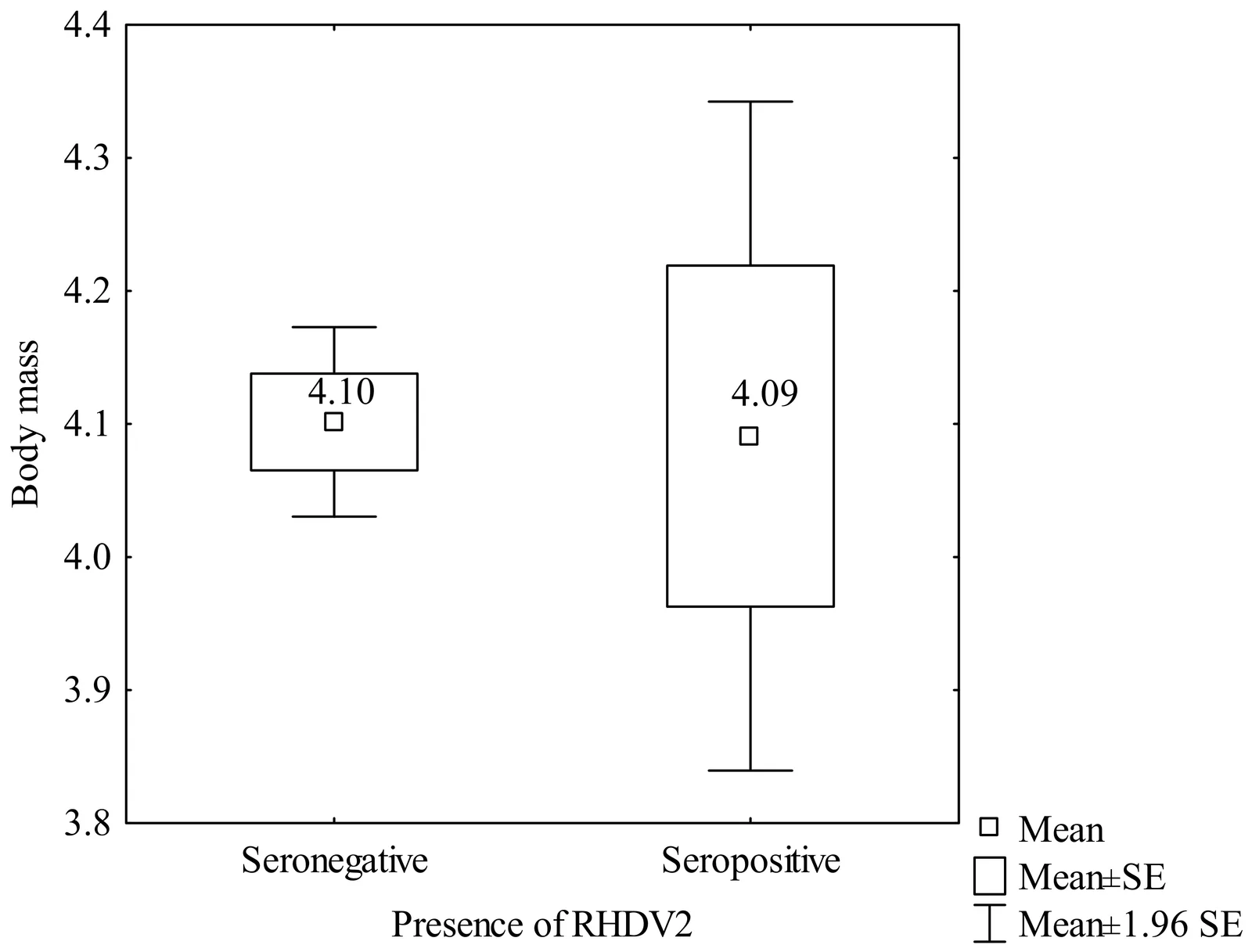
Body mass of hares according to their RHDV2 serological status (seronegative, seropositive). The small squares denote mean values, the boxes represent mean ± SE, and the whiskers denote mean ± 1.96 SE.

The observed RHDV2 seroprevalence was 11.5% in females (7/61; 95% CI: 4.8–22.2%) and 8.3% in males (4/48; 95% CI: 2.3–20.0%). No statistically significant association between sex and RHDV2 seroprevalence was detected (two-tailed Fisher’s exact test, *p* = 0.752; chi-square test, χ^2^ = 0.29, *p* = 0.589). Similarly, comparison of RHDV2-reactive antibodies titres between males and females using the Mann–Whitney U test revealed no statistically significant difference (U = 420.0; Z = −0.27; *p* = 0.611). No statistically significant association between age group and RHDV2 seroprevalence was detected (two-tailed Fisher’s exact test, *p* = 0.522; chi-square test, χ^2^ = 0.76, *p* = 0.382). Comparison of RHDV2-reactive antibodies titres between juvenile and adult hares using the Mann–Whitney U test also revealed no statistically significant difference (U = 1383.0; Z = −0.77; *p* = 0.442). Analysis according to the animals’ area of origin detected no statistically significant association between study area and RHDV2 seroprevalence (chi-square test, χ^2^ = 4.09; *p* = 0.394). Comparison of RHDV2-reactive antibodies titres among the study areas using the Kruskal–Wallis test also revealed no statistically significant differences (H = 4.04; *p* = 0.400). A comparison of hare body mass according to RHDV2 serostatus (Figure 5) showed mean body masses of 4.10 kg in seronegative individuals and 4.09 kg in seropositive individuals. No statistically significant difference in body mass was detected between these groups (t = 0.091; *p* = 0.927).

In order to identify the factors affecting the presence of EBHSV antibodies, a generalised mixed-effects model (ANCOVA) was used, in which the region of origin was included as a random effect, sex and age as fixed effects, and the individual’s body mass as a covariate. The only factor showing a statistically significant effect on the presence of EBHSV antibodies was body mass (*p* = 0.0061). Neither age (*p* = 0.1208) nor the random effect of the area (*p* = 0.2174) showed a significant effect on the serological status of hares, while sex showed a weak trend towards significance (*p* = 0.0777), with a higher seroprevalence recorded in females. In turn, an analysis of factors affecting the presence of RHDV2-reactive antibodies, considering EBHSV serological status, showed that the only statistically significant predictor of the presence of RHDV2-reactive antibodies was EBHSV serological status (*p* = 0.0366). Hares with antibodies against EBHSV exhibited a significantly higher prevalence of RHDV2-reactive antibodies. The other variables analysed, including age (*p* = 0.1609), sex (*p* = 0.5661), body mass (*p* = 0.9241) and the random effect of the sampling area (*p* = 0.7072), did not have a significant effect on RHDV2 seropositivity.

Given that EBHSV is the dominant serotype in the population (78.9%), individuals seropositive for RHDV2 are almost exclusively derived from a group of hares that already possess antibodies against EBHSV. This correlation strongly supports the hypothesis of cross-reactivity between the antigens of the two viruses (owing to their close taxonomic relationship within the *Caliciviridae* family) or points to an overlap in transmission routes and the presence of common environmental vectors that transmit both pathogens. In light of this, the overall assessment of serological data must also take into account the limitations inherent in diagnostic tests—including those using specific monoclonal antibodies—arising from the antigenic similarity between these two lagoviruses.

## 4. Discussion

The dramatic decline of the hare population in Poland since the 1970s is a long-standing phenomenon [58,59] that is worsening given the current ecological and population situation [11,60,61]. This situation is not an isolated one and reflects a much wider phenomenon occurring throughout Europe. This unfavourable development resulted from a number of factors: environmental factors (the use of chemicals on fields, changes in crop structure), factors linked to the urbanisation of rural areas (a reduction in arable land, changes in crop structure), and the occurrence of parasitic and infectious diseases [2]. Among the latter, the European brown hare syndrome (EBHSV), a fatal viral disease caused by the lagovirus EBHSV (GII.1), deserves mention first and foremost. A significant contribution of EBHS to the decline in hare populations has been observed since the 1980s, initially on the Scandinavian Peninsula, and later in most European countries [14,28]. In Poland, the impact of EBHSV on the hare population has been particularly pronounced since the mid-1990s [41].

The results from the present study regarding population indicators confirm earlier findings from studies conducted in four regions of south-eastern Poland, namely that body mass is determined solely by age, while the region of origin plays a lesser role. Body mass is a precise indicator of age and level of physical development in individuals, which means that heavier hares spend more time in the wild and are more likely to be exposed to the EBHS virus, a factor associated with the development of a humoral immune response. Greater body mass may also indicate good overall body condition in animals that have successfully survived infection and developed long-lasting population immunity. In terms of sex structure, females also predominated. However, the present study showed a balanced age distribution, with a slight predominance of juvenile individuals. As previously noted, key parameters related to reproduction and long-term population persistence indicate that, despite population densities that permit hunting, these populations are on the verge of being unable to continue functioning optimally [61]. These findings also confirm data from other studies in south-eastern Poland regarding body mass, but do not confirm the data on age structure [60]. In turn, a nationwide study conducted in 2009 also revealed differences in body mass only by age group, with females clearly predominating within each population. The average body mass was similar to the current figure reported by Misiorowska et al. [11]. Similar results regarding body mass were obtained in Hungary, with a slight advantage for juvenile hares over adults [5]. A Slovak study conducted in 2011–2012 in the Nitra region revealed a similar proportion of males and females in the population, although differences were observed in age groups and between study seasons [4]. At present, the body mass of hares in Poland is also similar to that in the Central Pomerania region [6], and significantly higher than that reported for the Greater Poland region [62] and north-eastern Croatia [3]. The results of a study into the body mass of continental and island hares in Sweden showed that hares living on the mainland had a higher body mass. There were no significant differences between the sex groups, while differences were found between juvenile and adult hares [63]. Body mass can also be correlated with climatic conditions. A study comparing adult female hares from Belgium (temperate oceanic climate) and Lower Austria (temperate continental climate) showed that the animals from Belgium had a higher body mass (4.3 kg) than those from Austria (3.4 kg) [64]. At present, the rates of reproductive dynamics and thus of population growth, although for the most part remaining at a level that enables continued slow population growth, are lower than those recorded in previous years in Poland, Croatia and Hungary [3,5,61]. Although the estimated population growth rates were positive, the relatively low reproduction coefficient, fluctuating around 1.0 in most study areas, suggests limited reproductive potential. Such values may reduce the capacity of local populations to compensate for additional mortality associated with environmental, anthropogenic, or infectious pressures. However, given the limited sample sizes in some study areas and the cross-sectional nature of the present study, these findings should be interpreted with caution and do not allow predictions regarding future population trends.

In recent years, type 2 rabbit haemorrhagic disease (RHD) has been identified as a potential threat to the hare population. RHDV2 (GI.2) is a lagovirus that is fatal to rabbits. This virus first appeared in France in late 2010, whereas in Poland, it has been present since 2016. The many distinctive features characterizing RHDV2 include its ability to overcome immunity in rabbits vaccinated against RHDV (GI.1), the mortality of juvenile rabbits under two months of age and, most importantly, its ability to infect hares and cause a disease similar to EBHS [45,46]. As has been repeatedly demonstrated, RHDV2 is capable of overcoming species barriers and occasionally infecting hares [48,49,56,65,66,67]. Although RHDV2 infection has not yet been confirmed in native European brown hare populations in Poland, the potential role of domestic rabbits, particularly those kept in traditional outdoor farming systems in rural areas, as a possible source of exposure warrants further investigation. However, the present serological findings do not allow the identification of potential sources or pathways of RHDV2 transmission.

The opportunity to obtain blood samples from hunted hares for serological testing enabled assessment of their serological status regarding EBHSV. The authors’ previous study, which was limited to two hunting areas in eastern and central Poland, revealed a high proportion of EBHSV-seropositive hares in those areas.

The present study, conducted on a sample of 109 hares, was expanded to include samples from new hunting areas, previously unexplored in this respect, and located along the eastern border of Poland and in the southern and central parts of the country (Figure 1). The results of EBHSV antibody tests showed a high seroprevalence of EBHSV-specific antibodies (78.9%) across all areas analysed. In a habitat located in the hunting area that was examined again after more than a year (Area I), the proportion of hares seropositive for EBHSV (72.3%) was similar to the level (75–90%) noted in the previous study [53]. Similar to the previous study, the overall proportion of EBHSV-seropositive hares was higher in females than in males (47.7% vs. 31.2%). However, with regard to age, a high proportion of seropositive hares was found in both juveniles (81.0%) and adults (76.1%), although these differences were not statistically significant.

The high proportion of EBHSV-seropositive hares, comparable to findings from a previous study [53] and recent reports from Italy [57], suggests widespread previous exposure to EBHSV among the examined animals. EBHSV antibody titres were generally low (10–40), reaching a maximum of 160 in only four hares. However, the antibody titer alone does not allow for an accurate determination of the time of exposure or the level of protective immunity.

The positive RHDV2 serology result found in 11 hares (10% of the total) previously diagnosed as EBHSV-positive (Figure 4) may indicate exposure to lagoviruses. An analysis of antibody titres revealed that, in 9 of the 11 hares with RHDV2-reactive antibodies, EBHSV antibody titres were higher than those measured for RHDV2, suggesting that cross-reactivity between the two lagoviruses cannot be excluded. In 6 serum samples, the ratio of EBHSV titre to RHDV2 (Rt ratio according to Velarde [56] ranged from 2 to 8, while in one sample, it was 1. However, in this case, exposure of the hares to RHDV2 cannot be ruled out, as the data may also result from a cross-reaction due to antigenic similarity, as noted by Estruch et al. [57].

A completely different serological response was observed for two hares from a habitat near Dąbrowa Tarnowska (Area V). In these cases, the RHDV2-reactive antibody titre (80) was 8 times and 4 times higher than the EBHSV antibody titre. Although these findings may be consistent with previous exposure to RHDV2, cross-reactivity cannot be definitively excluded. The presence of RHDV2 in this region is not unexpected. RHDV2 strains belonging to the GI.3/GI.2 genetic group were identified on several occasions in the study area between 2018 and 2020 [68], and more recently in 2025 (unpublished data). Moreover, the RHD outbreaks caused by RHDV2, recently detected for the first time in eastern Slovakia, have confirmed an epizootic situation in this European area, linked to the active circulation of GI.2 strains in the immediate vicinity of southeastern Poland [69]. This finding is particularly relevant in areas where RHDV2 has previously been detected in rabbit populations, including regions with small-scale farms operating under open rearing systems. Such epidemiological circumstances provide relevant context for interpreting RHDV2 seroreactivity in free-ranging hares but do not allow the source or direction of potential virus transmission to be determined. The detection of RHDV2-reactive antibodies in wild hares therefore represents an additional epidemiological observation whose significance for the health and population dynamics of this species remains to be determined.

It is difficult to state definitively whether the RHDV2-reactive antibodies observed in this study reflect prior exposure to RHDV2 or result from cross-reactivity due to antigenic similarities between EBHSV and RHDV2. Notably, higher antibody titers against EBHSV were not always accompanied by the presence of detectable RHDV2-reactive antibodies. For instance, one hare from Area I, which exhibited a relatively high antibody titer against EBHSV (160), showed no detectable RHDV2-reactive antibodies. Conversely, in some individuals—particularly those from Area V—RHDV2-reactive antibody titers were higher than those against EBHSV. While these findings might suggest prior exposure to RHDV2, cross-reactivity cannot be ruled out; therefore, these results should be interpreted with caution. These findings suggest that the detection of antibodies reactive to both viruses may have a complex basis, potentially reflecting antigenic similarity and cross-reactivity between EBHSV and RHDV2, as well as the documented occurrence of RHDV2 in the local epidemiological environment. Therefore, the RHDV2 serological findings should be interpreted with caution and require confirmation through molecular detection. Mixed-effects models were fitted using the full dataset (N = 109), to maintain statistical stability and robust degrees of freedom. However, the limited number of RHDV2-positive individuals (*n* = 11) necessitates cautious interpretation of the results. Consequently, multivariable findings for RHDV2 must be treated as exploratory trends rather than conclusive evidence of associations and require confirmation in larger studies.

## 5. Conclusions

Analysis of population indicators in brown hares (*Lepus europaeus*) from five regions of Poland showed comparable body mass across the study areas, although significant differences in this parameter were observed between juvenile and adult individuals. The estimated population growth indices were positive; however, the relatively low reproductive indices suggest limited reproductive potential in the studied populations. These findings should be interpreted with caution due to the small sample sizes in some study areas and the potential influence of regional and seasonal variation. Serological analyses demonstrated the presence of EBHSV-specific antibodies in hares from all five studied regions of Poland, which were characterised by stable population densities. The overall EBHSV seroprevalence was 78.9%, indicating widespread previous exposure of the examined hares to this virus. RHDV2-reactive antibodies were detected in 10.1% of the animals, providing evidence of serological reactivity to RHDV2 in free-ranging brown hares. Given the possibility of cross-reactivity between antigenically related lagoviruses, the RHDV2 serological findings should be interpreted with caution and require verification using molecular methods.

Combining the assessment of population indicators with serological data enabled a more comprehensive characterisation of the studied brown hare populations, encompassing population structure, reproductive indicators, and serological evidence of exposure to lagoviruses. The findings support the value of future integrated monitoring combining population assessments and serological surveillance with molecular diagnostics and genomic characterisation of lagoviruses. Such an approach could help clarify the epidemiological significance of EBHSV and RHDV2 and improve our understanding of lagovirus–host interactions in free-ranging *Lepus europaeus* populations.

## Figures and Tables

**Table 1:** Hare population indicators in the research areas.

Research Area	N	Body Mass (kg)		W_r_ Coefficient of Reproduction	W_sr_ Reproductive Index/Success Rate	W_pp_ Coefficient of Population Increase	W_p_ Coefficient of Population Growth
		Juvenile	Adult				
I	47	3.94	4.48	1.61	3.22	1.83	82.80
II	13	3.96	4.55	0.63	1.25	1.14	13.82
III *	12	-	4.18	-	-	-	-
IV	14	3.73	4.36	1.00	1.17	1.40	40.00
V	23	3.84	4.70	0.96	22.00	1.37	1527.90 **

* all the harvested hares are adult individuals. ** The extremely high population growth value (W_p_) in Region V reflects a hunting bag sampling bias (95.7% juveniles) rather than an actual biological rate of recruitment.

**Table 2:** Prevalence of antibodies to EBHSV in brown hares (*Lepus europaeus*) in the south, south-eastern, eastern and central regions of Poland from November to December 2022 (EBHSV ELISA serological).

Region of Origin	No of Tested Hares	Positive								Negative	
		No	Percentage ^1^	Anti-EBHSV Antibody Titre Distribution ^2^ Titre (Pab ELISA/Mab ELISA)						No	Percentage ^1^ %
				>10–40		>40–160		>160–640		<10	
				No	%	No	%	No	%	No	%
I	47	34	72.3	17	50.0	17	50.0	0	0	13	27.7
II	13	11	84.6	5	45.5	6	54.5	0	0	2	15.4
III	12	9	75.0	3	33.3	6	66.7	0	0	3	25.0
IV	14	12	85.7	5	41.7	9	58.3	0	0	2	14.3
V	23	20	87.0	8	40.0	12	60.0	0	0	3	13.0
Total	109	86	78.9%	38	44.2%	48	55.8%	0	0.0%	23	21.1%

^1^ percentage of positive/negative in relation to the total number of hares tested. ^2^ percentage of positive sera with the specified titre in relation to the total number of positives.

**Table 3:** Prevalence of RHDV2-reactive antibodies in brown hares (*Lepus europaeus*) in the south, south-eastern, eastern and central regions of Poland from November to December 2022.

Region of Origin	No of Tested Hares	Positive								Negative	
		No	Percentage ^1^	Antibodies Reactive to RHDV2 Titre Distribution ^2^ Titre (Pab ELISA/Mab ELISA)						No	Percentage ^1^ %
				>10–40		>40–160		>160–640		<10	
				No	%	No	%	No	%	No	%
I	47	3	6.4	2	66.7	1	33.3	0	0	44	93.6
II	13	1	7.7	1	100	0	0	0	0	12	92.3
III	12	3	25	3	100	0	0	0	0	9	75.0
IV	14	1	7.1	1	100	0	0	0	0	13	92.9
V	23	3	13.0	1	33.3	2	66.7	0	0	20	87.0
Total	109	11	10.1%	8	72.7%	3	27.3%	0	0	98	89.9%

^1^ percentage of positive/negative in relation to the total number of hares tested. ^2^ percentage of positive sera with the specified titre in relation to the total number of positives.

## Data Availability

The data presented in this study are available on request from the corresponding author.
